# Anaerobic sulfide removal involves an intricate interplay between biomass, biosulfur, and solutes

**DOI:** 10.1039/d5ew00927h

**Published:** 2026-01-27

**Authors:** Rikke Linssen, Sanne de Smit, Annemiek ter Heijne

**Affiliations:** a Environmental Technology, Wageningen University P.O. Box 17, Bornse Weilanden 9, 6708 WG, Building Axis z, building nr. 118 6700 AA Wageningen The Netherlands Annemiek.terHeijne@wur.nl

## Abstract

In the biodesulfurisation process harmful sulfide is converted to sulfur by sulfide oxidising bacteria (SOB), using oxygen as terminal electron acceptor. Surprisingly, in this process sulfide is already removed before oxygen is consumed. Therefore, sulfide and/or charge is being shuttled between sulfide removal and terminal electron transfer. Previously, it was thought that the bacteria themselves were the exclusive “electron shuttlers”. Patterns in sulfide concentration and oxygen reduction potential (ORP) during anaerobic sulfide removal tests in batch confirmed that SOB remove sulfide in two steps, of which the second lowered the ORP. However, we found that aside from biomass also biosulfur and certain solutes are involved in electron shuttling. Gradual removal of sulfide by interactions between sulfide, solutes, and biosulfur caused an increase in ORP, even after all sulfide was removed. The amplitude and rate of ORP increase rose with increasing sulfide removal capacity of the process solution. We hypothesise that organic thiol/disulfide redox couples are involved in electron shuttling.

## Abstract 2

In the biodesulfurisation process sulfide removal starts in the anaerobic section where biomass, organic solutes, and biosulfur together bind and transport sulfide equivalents to be oxidized in the aerobic reactor, thereby acting as sulfide shuttles.

## Graphical abstract

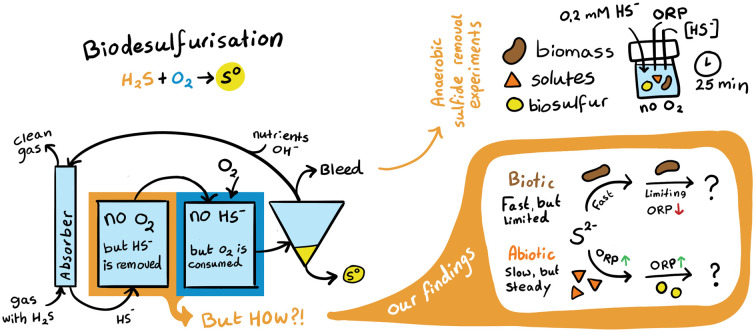

**Water impact**

Our work shows that sulfide oxidising bacteria active in biodesulfurisation processes are able to produce organics that can anaerobically remove sulfide, possibly acting as redox mediator. Better understanding of the biodesulfurisation process facilitates more efficient process design and control. Furthermore, these findings open up possibilities of producing energy storage compounds from sulfur-containing waste for development of renewable energy storage technologies.

## 1 Introduction

### 1.1 Biodesulfurisation as sulfur remediation technology

**Fig. 1:**
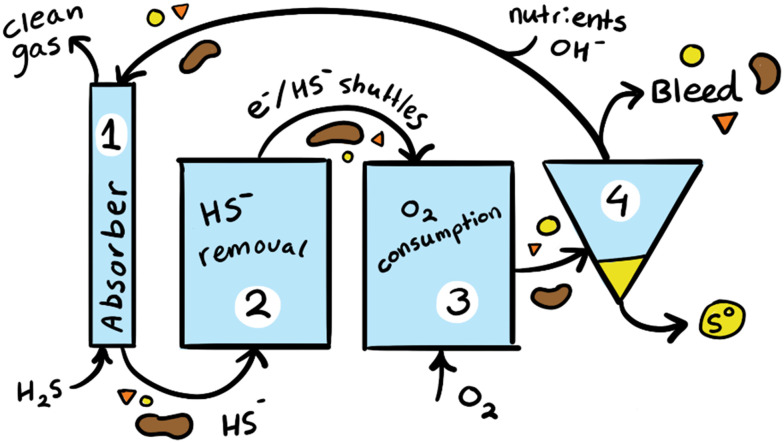
Schematic representation of the biodesulfurisation process containing an absorber column (1), anaerobic reactor (2), aerobic reactor (3), and decanter (4).

Due to its toxicity and impact on the environment sulfide needs to be removed from waste streams before they are deposited in the environment. A relatively environmentally friendly way of removing sulfide from gas or liquid waste is by biological desulfurisation. The biodesulfurisation process consists of four units (Fig. 1). In the absorber column (1) sulfide containing gas is absorbed into the haloalkaline process solution. The sulfide containing solution then flows to the anaerobic reactor (2) where it is exposed to extended time in anaerobic conditions. Then in the aerobic reactor (3) sulfide oxidising bacteria (SOB) convert sulfide to sulfur using oxygen as terminal electron acceptor. The resulting sulfur crystals are removed using a decanter (4) and the process solution minus a small bleed stream is recirculated to the absorber column.

The extended exposure time to anaerobic conditions provided in the anaerobic reactor is beneficial for the biodesulfurisation process: it increases selectivity for production of sulfur from 75 to 97%, reduces formation of side products such as thiosulfate and sulfate by 90%, lowers the need for caustic addition,^1^ increases the settleability of the produced sulfur particles, and helps preventing the formation of foam.^2^ These beneficial effects have been suggested to be caused (partly) by so called ‘electron shuttling’.

### 1.2 Electron shuttling

In the anaerobic reactor of the biodesulfurisation process as described above the sulfide concentrations are much lower than expected, up to half of the theoretical sulfide concentration, despite the absence of oxygen as electron acceptor.^3^ Nevertheless, the oxygen consumption in the aerobic reactor matches the sulfide influx in the adsorber column. This indicates that sulfide removal and oxygen consumption are spatially separated between the anaerobic and aerobic reactor, and that electrons and/or sulfide are being shuttled between sulfidic and aerobic conditions. So far several works have been published by our group on investigating electron shuttling,^4–10^ but its underlying mechanisms are still not fully understood.

Sulfur is an essential element for (micro)biological growth and therefore always required in microbial processes. On the one hand the presence of sulfur species sometimes poses problems. In microbial fuel cells for example, sulfide can oxidise towards solid sulfur at the electrode surface which decreases the active electrode area, thereby hindering cell performance.^11,12^ At the same time, this sulfur deposition could form a basis for novel sulfur-based energy storage technologies.^13^ In both cases electron shuttling could be used, either as a strategy to prevent sulfur deposition on electrodes or to store (electrical) energy. So far, the understanding of interactions between sulfur species and microorganisms is limited. Thus, a better understanding of microbial sulfur interactions in terms of electron transfer and energy storage will, besides increasing the efficiency of biological desulfurisation process, aid towards the development of novel sustainable biobased technologies.

### 1.3 Let's not forget about biosulfur and solutes

To research electron shuttling, previous work has focused on measuring anaerobic sulfide removal and terminal electron transfer of SOB biomass that was separated from the process solution using centrifugation and washing steps. This was done to prevent interference from sulfur particles and solutes present in the process solution. These tests showed that in the presence of biomass sulfide is gradually removed, that anaerobic sulfide removal can, at least partly, be allocated to activity of SOB,^5,7^ and that polysulfides are produced during the process.^6^ However, sulfide removal in the presence of only biomass was limited and has not been able to fully explain sulfide removal observed in the bioreactor setup.

Thus, we believe that aside from biomass also the other components in the process solution, namely sulfur particles and certain solutes, are involved in anaerobic sulfide removal. To test this hypothesis we characterised the sulfide removal behaviour of SOB biomass, sulfur particles, and the solutes, individually and in combinations. We compared process solutions from several biodesulfurisation setups with varying electron shuttling activities to get more insight in which components are determining sulfide removal rates. Also new in this work is that aside from sulfide concentration we measured the oxygen reduction potential (ORP) in time, by which various rate limiting steps were uncovered even after sulfide removal stopped.

## 2 Materials and methods

### 2.1 General

In this work, whenever the term “sulfide” is used, this indicates dissolved sulfide, including HS^−^, S^2−^ and H_2_S, and polysulfides measurable by the methylene blue assay described in 2.4. The term ‘sulfide removal’ is used to nominate the decline in measured sulfide concentration.

Stock solutions of sulfide (NaSH, Acros Organics, Belgium) were prepared in batch bottles using demineralised water that was made anaerobic by flushing with nitrogen gas. The total sulfide content was then measured using redox titration with AgNO_3_ (Titrino Plus, Metrohm, Switzerland). In a mixture of 80 ml 5% NaOH and 10 ml 2.5% NH_3_, 0.1 ml sample was titrated with 0.1 M AgNO_3_ in triplicate. All experimental work involving sulfide was performed wearing a personal H_2_S monitor (H_2_S sensor Pac 6500, Dräger, Germany).

### 2.2 Effluent pre-treatment

Bleed, also called effluent, was collected from three different biodesulfurisation setups. Eerbeek effluent was harvested from a full-scale single-reactor biodesulfurisation plant stationed at the municipal wastewater treatment plant in Eerbeek in June 2023 (Industriewater Eerbeek BV, The Netherlands). The setup consists of an absorber column, an aerobic reactor, and a decanter, but no anaerobic reactor (a single-reactor setup). The setup treats 4000–16 000 m^3^ gas per day at a sulfide concentration ranging between 4000 and 16 000 ppmv,^14^ which comes down to a sulfide load of 20–350 kg per day. WUR effluent was collected from a pilot-scale biodesulfurisation plant stationed at Wageningen University^3^ in March 2022 (WUR-3), in December 2023 (WUR-1), and in February 2024 (WUR-2). The pilot setup was configured as a dual-reactor setup and contained an absorber of 4 m (HRT 5–15 min), an anaerobic reactor of 6 L (HRT 10–30 min), and an aerobic reactor of 11.4 L (HRT 30–60 min). The setup handled a maximal sulfide load of 170 gr per day. Wetsus effluent was harvested from a lab-scale dual-reactor biodesulfurisation setup stationed at Wetsus, Leeuwarden in October 2022.^15^ The reactor contained an absorber column of 70 ml, an anaerobic reactor of 2.2 L and an aerobic reactor of 3 L and a sulfide load up to 25.5 gr per day sulfide.

**Fig. 2:**
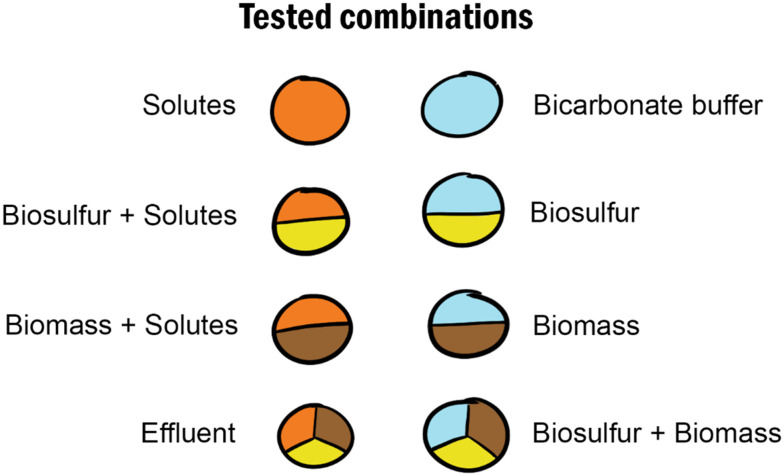
Combinations of components of biodesulphurisation effluent tested in this work.

The effluent was aerated overnight to oxidise remaining (poly)sulfide to sulfur and sulfate, and to oxidise the internal redox pool of the biomass. Effluent was then tested or further treated to produce different effluent fractions: biomass, biosulfur, and the solutes (Fig. 2). These fractions were produced by centrifuging the effluent 30 minutes at 7500×*g* (High speed centrifuge Z 36 HK, Hermle LaborTechnik, Germany). The supernatant was collected and pushed through 0.2 and 0.2 μm filter (Chromafil Xtra PES-20/25, Macherey-Nagel, Germany) to produce the **solutes**. Centrifugation (15 minutes at 15.000 rpm, Centrifuge 5425, Eppendorf, Germany) confirmed the absence of particles in the solutes. The brown top layer of the pellet was carefully resuspended in bicarbonate buffer (1 M NaHCO_3_, pH 8–8.5, EMSURE Merck, Germany) keeping most of the white-yellow sulfur layer on the bottom of the pellet intact. The biomass suspension was then cleaned by repeating centrifugation and washing steps until it contained no more sulfur, producing the **biomass** fraction. After the last washing step, the biomass was aerated overnight to remove residual sulfur and to oxidise the internal redox pool.^4^ The remaining biosulfur was resuspended in bicarbonate buffer, producing the **biosulfur** fraction. **Combinations** of these fractions were made by resuspending the biomass or biosulfur in the solutes and by resuspending biomass and biosulfur together in bicarbonate buffer (Fig. 2). After pretreatment all samples were stored at 4 °C up to a week.

### 2.3 Sulfide removal experiments

Experiments were performed in 70 ml reactors with 50 ml liquid volume in an anaerobic tent (2 L zip lock bags, HEMA, The Netherlands) where the atmosphere was flushed with nitrogen. Before sulfide addition, the reactor content was made anaerobic by flushing with nitrogen gas and the pH was measured. After sulfide was added, 2 mL samples were taken from the liquid periodically in a timeframe of 25–30 minutes to measure the sulfide concentration. This timeframe reflects the hydraulic retention time (HRT) of the anaerobic reactor in the dual-reactor setup.^3^ Control tests were performed on bicarbonate buffer and Table 1 contains an overview of all tested effluents, fractions or combinations, and number of experimental replicates. In the result section the individual sulfide concentration measurements of the replicates are shown. For the ORP, the moving average is shown of the pooled measurements from the replicates.

**Table 1:** Effluent fractions tested during the ORP experiment. Sulfide load was 0.2 mM. The number in brackets shows the number of replicates

Tag	Location setup	Fractions and combinations tested (replicates)
PS-1	WUR pilot scale	Effluent (2), biomass (3), solutes (2), biomass + solutes (5)
PS-2		Effluent (4), biomass + biosulfur (2), solutes + biosulfur (2), solutes (4)
PS-3		Effluent (2), biomass (2), solutes (2)
LS	Wetsus lab scale	Effluent (5), biomass (4), solutes (6)
FS	Eerbeek full scale	Effluent (2), biomass (2), solutes (4)
—	Abiotic	Bicarbonate buffer (5), biosulfur (3), medium (4)^a^

a Medium consisted of bicarbonate buffer with a mix of nutrients and trace elements as described by Johnston *et al.* (2023).^9^

The oxidation reduction potential (ORP) of sulfidic solutions was measured using a ORP probe (Redox electrode QR400X/6 mm, ProSense, The Netherlands) connected to a ORP measuring device (C3010 multi-parameter analyser, Prosense, The Netherlands). Data was collected through LabView on a PC. The oxygen reduction potential (ORP) measures the tendency of the solution to either oxidise or reduce and can be expressed similar to the Nernst equation (eqn (1)), where *E*^0^_H_ is the reference potential, *R* is the gas constant, *T* is temperature in Kelvin, *n* is the number of electrons involved, *F* is the Faraday constant, and Π_ox_ and Π_red_ are the sum of oxidation or reduction species in the solution.1
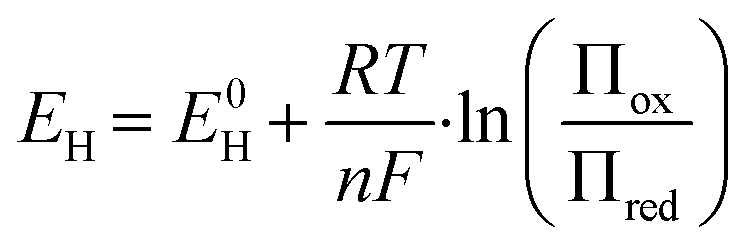
ORP can be used as an indication of the ratio between electron acceptors and electron donors, where a higher ORP value indicates the presence of stronger electron acceptors, and a lower ORP indicates the presence of stronger electron donors such as sulfide. Aside from electron donors and acceptors, the ORP is also sensitive to change in pH, temperature, and salinity. However, in solutions that are not in equilibrium, the ORP is mainly dependent on compounds with the highest electron exchange rate with the sensor.^16^ During sulfide removal experiments ORP is measured as an indication for changes in the composition of the sample solution.

### 2.4 Analysis

Sulfide was measured by a colorimetric assay based on methylene blue (LCK 653, Hach Lange, USA). Samples taken from the reactor were filtered through a 0.2 μm filter and diluted at least 2 times with 0.1 M ZnAcetate directly in the Hach Lange tube.^5^ The methylene blue assay measures the total dissolved sulfide as H_2_S after acidification of the sample with sulfuric acid. Organosulpur compounds such as methanethiol, ethanethiol, propanethiol, and dimethyl disulfide are not detectable with this method and do not interfere with the total sulfide measurement.^17^ The lowest detection limit of the methylene blue assay was 0.006 mM sulfide. If no colouration was observed, the absence of sulfide was confirmed by lead acetate paper (MQuant, Supelco, USA).

The biomass concentration was expressed as mgN L^−1^ (LCK 138, Hach Lange, USA).^3^ Total organic carbon (TOC) was measured at 10 and/or 20× dilution (TOC-L CPH/CPN, Shimadzu, Japan). DNA was concentrated by passing 20–50 mL of solutes trough a DNA-filter (0.22 μm PVDF membrane, Merck Millipore, Ireland) DNA extraction was done using DNeazy power soil pro kit (Qiagen, Germany). The final DNA concentration was measured (Qubit 1× dsDNA HS Assay Kit & Qubit 4 fluorometer, ThermoFisher Scientific, USA).

New generation sequencing was performed on the DNA samples. DNA samples of PS-1, PS-3, and LS were processed as described in Smit *et al.* using SILVA reference database to pick OTUs and classify the sequences.^18^

### 2.5 Bimane extraction

For the bimane extraction PS-1 effluent was used. Biomass and biomass combined with the solutes were prepared according to 2.2. The presence of dissolved thiols was measured using a bimane extraction method derived from Rodriguez *et al.* (2011).^19^ Bimane extraction was performed in an anaerobic glove bag (Captair pyramid glove bag 22609CN, Erlab, France). Biomass suspension was made anaerobic by sparging with nitrogen gas. The solutes were then collected by pushing 10 ml biomass suspension through glass filters (GF/F *ø* 25 μm and 0.7 μm particle size, Whatman, UK) in a reusable filter holder (polypropylene filter holder for 25 mm membranes, Cole-Parmer, USA). Filtrant was fixated by pipetting 50 μL in 0.5 mL D-mix (9.4 mM monobromobimane, 50 mM HEPES, 5 mM EDTA in 50% v/v acetonitrile). Then, 2.0 mM sulfide was added to the biomass suspensions. After half an hour another sample was taken, pushed through the filter, and fixated with D-mix. The samples were stored in the dark at 4 °C before they were run on HPLC.

HPLC was performed on a reversed-phase-column (Alltech Prevail C18, 5 micron particle size, 150 × 4.5 mm), at 35 C at a flowrate of 1 ml min^−1^ on a Shimadzu LC40 system.^20^ The eluent system was: (A) acetic acid (0.25%, pH 4); (B) methanol (100%, v/v). The elution protocol was as follows: 0–7 min 88% A, 12% B isocratic, 7–15 min 12–30% B linear gradient, 15–19 min 30% B isocratic, 19–23 min 30–50% B linear gradient, 23–30 min 50–100% B linear gradient, 30–33 min 100% B isocratic, 33–33.1 min 100–12% B linear gradient, 33.1–36 min 12% B isocratic (column regeneration). A standard containing cysteine, glutathione, thiosulfate, coenzyme M (CoM), bimane, and sulfide was run. The amount of standard injected ranged from 3.9 to 1000 pmol (0.00 to 0.05 mM). Compounds with peaks below 50 V min were excluded from analysis.

## 3 Results and discussion

Sulfide removal experiments were performed on effluents from various sources, on separate effluent fractions, on several combinations of these fractions, and sometimes at different sulfide loads. This resulted in an elaborate dataset of sulfide concentration and ORP trends. For readability we'll focus on a selection of the data in the main text. The rest of the results can be found in the SI and include relative microbial community composition, sulfide and ORP measurements of all tested samples (averaged) also at varying sulfide loads, characteristics of and preliminary tests performed on effluent solutes, and a detailed overview of observed sulfide removal/ORP patterns.

**Fig. 3:**
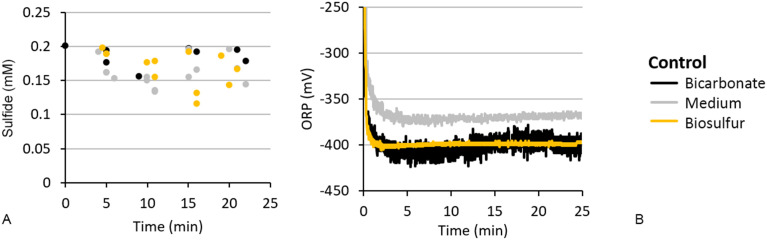
Abiotic controls showing (A) sulfide concentration and (B) ORP in time after dosing 0.2 mM sulfide in bicarbonate buffer (black), medium (grey) and bicarbonate buffer with sulfur particles (yellow).

As control we measured the sulfide concentration and ORP in bicarbonate buffer with and without trace elements or biosulfur particles (Fig. 3). After dosing 0.2 mM sulfide the measured sulfide concentration in the controls ranged between 0.15 and 0.2 mM, showing a slight underestimation and error of ∼0.02 mM. After sulfide addition the ORP decreased to −400 mV (to −370 mV when trace elements were present) within 2 minutes and stayed stable up to the end of the experiment.

### 3.1 Effluents vary in sulfide removal behaviour

**Fig. 4:**
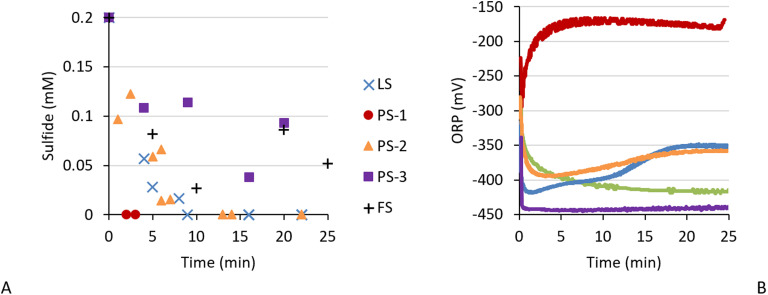
Sulfide concentration (A) and ORP (B) during anaerobic sulfide removal in effluent gathered from different biodesulfurisation setup: LS (blue, crosses), PS-1 (red, circles), PS-2 (orange, triangles), PS-3 (purple, squares), FS (green, plus signs). The sulfide load was 0.2 mM.

All effluents tested showed sulfide removal after adding 0.2 mM sulfide, but the sulfide removal rate and degree varied (Fig. 4A). Effluent FS from a single-reactor setup removed about 0.15 mM within the first five minutes, after which the sulfide concentration stagnated. Effluent PS-1, PS-2, and LS, which were sourced from dual-reactor setups, showed complete sulfide removal. It is known that dual-reactor setups show more electron shuttling than single-reactor setups,^4^ so these findings are not unexpected. But new is the detection of differences in sulfide removal rates. PS-1 completely removed all sulfide within 2–3 minutes, reaching a max. sulfide removal rate of 0.10 mM min^−1^, whereas sulfide removal in PS-2 and LS effluent took 10 minutes to complete at a rate of about 0.03 mM min^−1^. The difference between PS-1 and LS could be attributed to the difference in sulfide load, as the effluents were sourced from a setup with a sulfide load at 8 gr per day per L aerobic reactor for LS compared to 15 gr per day per L for PS-1. As high sulfide concentrations inhibit sulfide oxidation, electron shuttling could be a survival mechanism to lower the sulfidic pressure on the SOB. The higher sulfide load of PS-1 caused more sulfidic pressure and could have induced faster electron shuttling to counteract the higher sulfide load. PS-1 and PS-2 were harvested from the same setup, but during operation of PS-2 the standard sodium bicarbonate buffer was mixed with potassium carbonate. Microbial metabolism can responds differently to Na^+^ and K^+^,^21^ thus a change from sodium to potassium could have influenced microbial metabolism. PS-3 was also harvested from this setup, but after 20 minutes 0.05–0.1 mM sulfide remained in solution. Since the other effluent samples taken from this reactor setup were either directly processed (PS-1) or stored for up to a month (PS-2) this decline in sulfide removal capacity is attributed to long (1.5 year) storage time of the PS-3 effluent, which likely caused a decrease in biological activity and degradation of metabolites.

Thus, the sulfide removal varied and was incomplete (FS, PS-3), or complete at a slow rate of ∼0.03 mM min^−1^ (LS, PS-2) and a fast rate of max 0.10 mM min^−1^ (PS-1). To better understand the processes that occur during anaerobic sulfide removal we measured the ORP alongside of sulfide concentration (Fig. 4B). Here we see something interesting happening. The ORP in effluent with slow sulfide removal acted like the abiotic controls (Fig. 3): the ORP decreased −450 mV for PS-3 and to −420 mV for FS and then stabilised. However, for medium to fast sulfide removing effluent the ORP increased again after the initial drop in ORP caused by sulfide addition: in 20 minutes the ORP gradually increased in PS-2 effluent from −395 to −355 mV and in LS effluent from −420 to −350 mV. The ORP in the best sulfide remover (PS-1) rapidly increased in the first five minutes from −300 to −175 mV. Here we observe two points of interest:

#### 3.1.1 The effect of sulfide removal on ORP varied between microbial communities

For operating the desulfurisation process it is assumed that the ORP is controlled by sulfide load and oxygen supply. However, in previous research de Rink *et al.* observed that within five minutes after sulfide was added in anaerobic conditions the ORP stabilised at a higher ORP in the presence of biomass (−325 mV) compared to the abiotic control (−425 mV), thus it was concluded that aside from sulfide and oxygen also the biomass themselves influenced the ORP.^10^ They hypothesised that that different microbial communities could have a different effect on the ORP. To test this hypothesis we added the same reductive equivalent (*i.e.* sulfide) to five different microbial communities with varying compositions (see Fig. S1 for the community composition) and observed varying responses in terms of sulfide removal and ORP (Fig. 4), proving that the ORP responds differently to different microbial communities hypothesis.

Since the effect on the ORP varied between SOB communities in terms of rate and amplitude, which were both the highest for the community with fastest sulfide removal, we hypothesise that the effect on ORP increases in rate and amplitude with an increasing sulfide removal rate. This behaviour is specifically pronounced in PS-1, where the ORP became 225 mV higher (−175 mV) compared to the control (−400 mV). The increase in ORP after the initial drop caused by sulfide addition as observed in PS-1, PS-2, and LS indicate that reactive sulfide is converted to a less reactive state. This is further discussed in section 3.3.2 and 3.4.

#### 3.1.2 The ORP increase continued even after all sulfide was removed

After all sulfide was removed, at <2–3 min in PS-1 and at ∼10 min in PS-2 and LS, the ORP still increased. While for PS-1 it is difficult to say if this was caused by lag of the ORP sensor or not, as the ORP stabilised after 5 minutes, in PS-2 and LS the ORP kept increasing and only stabilised 10 minutes after all sulfide was removed. This indicates that sulfide removal is a multistep process where both sulfide removal and its follow up steps influence ORP. These sequential steps are most visible in LS effluent, where the ORP takes two increments: one from −420 to −400 mV which took place during sulfide removal up to 10 min, and one from 10 to 20 min which took place after all sulfide was removed. This phenomenon is further discussed in section 3.3.2.

### 3.2 Biomass removes sulfide in two subsequent steps

#### 3.2.1 Step 1: sulfide removal by biomass only partially explains sulfide removal in effluent

**Fig. 5:**
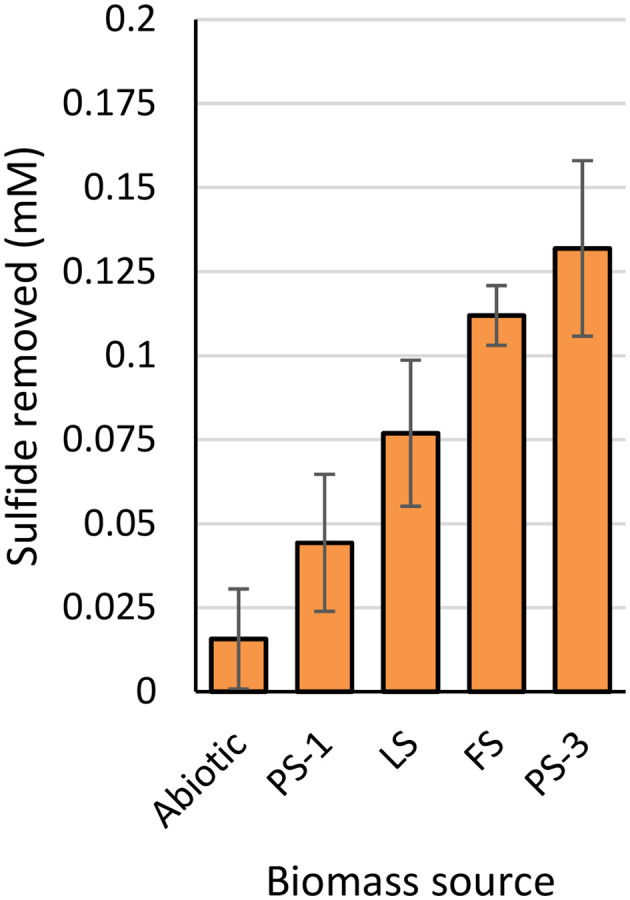
Amount of sulfide removed per biomass source after 5 minutes of incubation with 0.2 mM sulfide.

For all tested effluents, biomass resuspended in bicarbonate buffer could not completely remove the 0.2 mM sulfide that was dosed. The little sulfide removal that did take place happened within the first five minutes, after which the sulfide concentration stayed stable (Fig. S2C.1). The amount of sulfide removed varied between biomass source (Fig. 5). Biomass from PS-3 and FS removed about 0.1–0.15 mM sulfide, which is comparable to the sulfide removal observed in their effluent. By contrast biomass of LS only reached up to ∼0.075 mM sulfide removal, where full removal was obtained in the effluent. Interestingly, PS-1 biomass removed the least sulfide (∼0.04 mM) while in the effluent sulfide was completely removed in 2 minutes.

The biomass concentration in all effluents was comparable and ranged between 30 and 40 mgN L^−1^, the biomass concentration in the biomass samples is expected to be slightly lower due to losses of biomass during the washing steps. In earlier work we observed that the biomass concentration has limited effect on sulfide removal at concentrations higher than 3 mgN L^−1^. Therefore, the deviation in sulfide removal between biomass and effluent is not expected to be caused by differences in biomass concentration. The effluent where all sulfide removal can be attributed to biomass either came from a single-reactor full-scale setup (FS) or was very old and inactive (PS-3). Thus, whereas previous work assumed that electron shuttling observed in the anaerobic reactor of the PS setup was fully attributed to activity of SOB, it is now confirmed that aside from biomass also other components are needed to obtain full anaerobic sulfide removal.

#### 3.2.2 Step 2: slow enzymatic conversion influences ORP

**Fig. 6:**
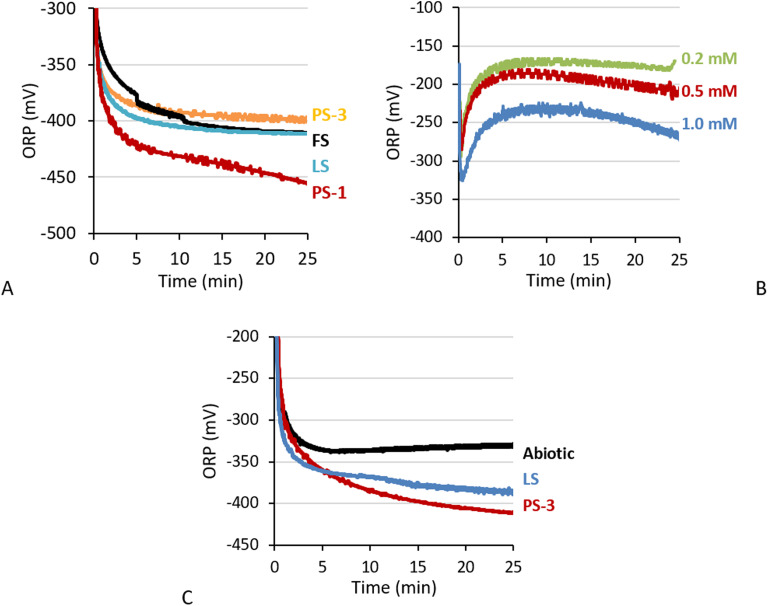
A) ORP during anaerobic sulfide removal of biomass at a sulfide load of 0.2 mM of PS-3 (orange), FS (black), LS (blue), and PS-1 (red). B) ORP during the sulfide removal experiment with PS-1 effluent. Sulfide dosing was 0.2 mM (green), 0.5 (red), and 1.0 mM (blue). C) ORP of LS (blue) and PS-3 (red) biomass or abiotic bicarbonate buffer (black) dosed with 0.1 mM sulfide.

Interestingly, in PS-1 biomass we saw a gradual decrease in ORP after 5 minutes from ∼−400 to ∼−420 mV (Fig. 6A). The gradual decrease in ORP after the sulfide concentration stabilised ORP visible in PS-1 effluent when dosed with 0.5 and 1 mM (Fig. 6B) and when dosing PS-2 and PS-3 biomass with 0.1 mM sulfide (Fig. 6C) (see Fig. S2C.1, S3A and S4A for corresponding sulfide concentrations), indicating that after sulfide removal an additional process took place that lowered the ORP. This is in contrast with earlier findings, which imply that electron shuttling SOB only increase ORP.^10^

Electron shuttling of SOB biomass has already been described in our previous works.^5,7^ Batch sulfide removal experiments at varying biomass concentrations, pH, and sulfide load showed that sulfide removal of biomass occurred in two sequential steps. We were able to mathematically describe the observed sulfide removal by a fast chemical process following a slower enzymatic process dependent on pH. The rapid removal step as described in 3.2.1 corresponds with the fast chemical equilibrium described in earlier work that is hypothesised to involve spontaneous cleavage of sulfur bonds in sulfur globules.^7^ Sulfide oxidising bacteria contain sulfur globules, either internal or attached to the exterior of the cell, which contain cyclooctasulfur (S_8_), polysulfides, persulfides, and polythionates.^22–25^ The internal polysulfide pool of SOB has a polysulfides with a chain length ranging between 2 and 8.^26^ Irreversible cleavage of these sulfur compounds by insertion of sulfide would result in an accumulation of sulfur in the globules. Calculations based on sulfur globule density estimated cells increase about 1.5–2 times at maximal sulfide removal capacity.^5^2
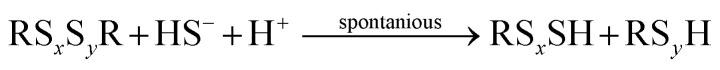
It is known the chain length in biodesulfurisation effluent is effected by the presence of SOB^9^ and that sulfide quinone reductase and flavocytochrome c, enzymes involved in sulfide respiration, are known to interact with polysulfides.^27,28^ Thus we hypothesise that during the slow ORP decrease observed in this work these enzymes slowly oxidise (eqn (3)) and recombine (eqn (4)) the smaller polysulfides formed in eqn (2) into one. The electrons released in eqn (3) could then be stored in intracellular or extracellular redox mediators. Investigating the polysulfide chain length distribution during sulfide removal could give more insight into this mechanism.3
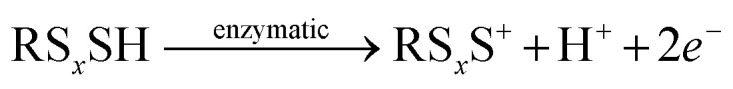
4
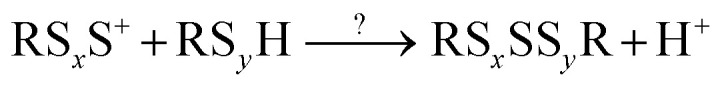


### 3.3 Solutes and biosulfur should not be neglected when determining electron shuttling

#### 3.3.1 Step 3: solutes are unexpected sulfide remover

**Fig. 7:**
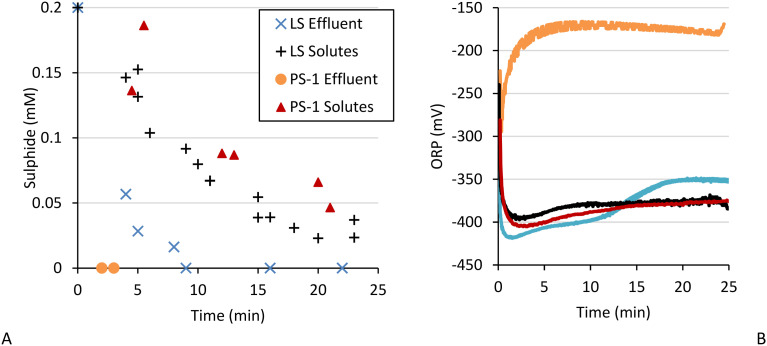
Sulfide concentration (A) and ORP (B) during sulfide removal in LS effluent (crosses, blue) and solutes (plusses, black) and PS-1 effluent (circles, orange) and solutes (triangles, red) at a sulfide load of 0.2 mM.

Aside from biomass we were able to separate two other effluent fractions: biosulfur and the solutes. No sulfide removal was observed in the solutes of FS, PS-2, and PS-3 (Fig. S2D.1). However, the solutes of PS-1 and LS effluent did show sulfide removal (Fig. 7A). Both for PS-1 and LS the sulfide removal by only solutes were not as fast as in the effluent. While the effluent saw complete sulfide removal after 2–3 minutes for PS-1 at a rate of max. 0.10 mM min^−1^ and after 10 minutes for LS at a rate of 0.025 mM min^−1^, in presence of only solutes sulfide was gradually removed at an initial rate of 0.013 mM min^−1^, reaching 0.02–0.05 mM after 25 minutes.

While the ORP in the effluent of PS-1 and LS varied greatly, the ORP in their solutes behaved similarly: after the initial drop to ∼−400 mV the ORP increased over the course of 7–10 minutes, stabilising at ∼−375 mV (Fig. 7B). This increase in ORP was comparable to the first ORP increment observed in LS effluent between 1 and 5 min. Since this increment was only observed in samples where solutes were present and sulfide was removed we hypothesise that this increment in ORP is related to processes underlying sulfide removal. Why the ORP stabilises in LS and PS-1 solutes before sulfide removal is finished is unclear. Possibly after 7–10 minutes sulfide removal is limited by another process that has similar rates but no effect on ORP.

It is fascinating how much and how quick sulfide was removed PS-1 and LS solutes, but it is quite strange how sulfide removal was not observed in the solutes of PS-2 while sulfide removal in the effluent in PS-2 and LS effluent was comparable. Also, the sum of sulfide removed in biomass and the solutes samples of PS-1 still not matched the seamingly instant sulfide removal observed in the effluent. Simply combining the solutes with aerated biomass did not increase sulfide removal, as observed in PS-1 (Fig. S5A). Therefore, there must have been another factor at play.

#### 3.3.2 Step 4: biosulfur as sulfide removal agent

**Fig. 8:**
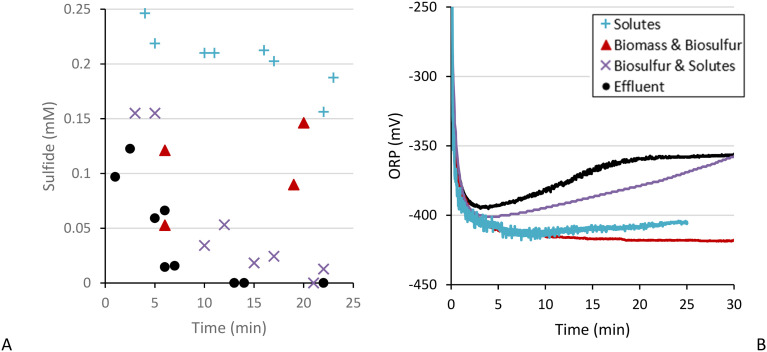
Sulfide concentration (A) and ORP (B) of fractions and combination of PS-2: solutes (blue, plusses), biosulfur & solutes (purple, crosses), biomass & biosulfur (red, triangles) and effluent (black, circles).

After removing the biomass and the solutes one fraction remained: biosulfur. We tested the sulfide removal in biosulfur resuspended in bicarbonate buffer and found that biosulfur on itself is not able to remove sulfide (Fig. 3), also combining biomass and biosulfur did not result in more sulfide removal than for biomass on its own (Fig. 8). But, after recombining the solutes of PS-2 with biosulfur directly after pretreatment we did see an effect. While PS-2 solutes on itself did not show sulfide removal, after adding biosulfur sulfide was gradually removed at an initial rate of 0.01 mM min^−1^ and almost complete within 25 minutes. The ORP also increased from −400 to −370 mV at 25 min. The sulfide removal and ORP increase was not as fast as observed in the effluent, which had a sulfide removal rate of about 0.03 mM min^−1^ and where the ORP reached −370 mV at 15 min, which hints that still biomass is required for maximal sulfide removal.

Furthermore, as mentioned in section 3.1.2, in PS-2 effluent the ORP increase continued even after all sulfide was removed. When biosulfur and the solutes were recombined this was also observed as the ORP kept increasing even though (almost) all sulfide was removed after 23 min (Fig. 8). This indicates the presence of another process, one where the intermediates formed by interaction between sulfide and solutes are further converted. Why in some cases solutes showed sulfide removal and in some cases the presence of biosulfur is required remains unclear and requires further identification of solutes and enzymatic activity.

### 3.4 Organic electron shuttlers

In all samples where we observed gradual and/or full sulfide removal solutes were present, even though in the case of LS solutes the presence of sulfur was required. In samples with biomass and/or biosulfur where solutes were absent either no sulfide removal was observed or sulfide removal reached its limit within the first five minutes. Thus, we conclude that solutes act as a key player in anaerobic sulfide removal.

Now we know that certain compounds in the solutes interact with and remove sulfide, but not which compound is responsible for sulfide removal. Biodesulfurisation setups are fed with a bicarbonate buffer containing trace elements which could chemically react with sulfide to metal sulfides. Therefore we tested bicarbonate buffer containing trace elements for sulfide removal. Since no sulfide removal was observed we conclude that oxidation of sulfide with trace metals as terminal electron acceptor did not contribute to sulfide removal (Fig. 3).

To explore the content of the solutes we measured several characteristics of the solute samples, such as pH, total nitrogen, DNA content, organic carbon content (Table 2). Furthermore, we tried COD, ICP, and NMR to identify or characterise the content of the solutes, but the methods available to us were not suitable for producing quality data (SI A). No clear link was found between sulfide removal and any of the measured characteristics, but N/C ratio is able to tell us something about the nature of the dissolved organics: the total nitrogen content ranged between <1 and 6 mgN L^−1^ and the total organic carbon content between 22 and 57 mgC L^−1^. The ratio between nitrogen and carbon thus ranged between 0.01 and 0.15 N/C molar ratio, whereas biomass is often represented with a N/C ratio of 0.2. This indicates the presence of non-nitrogenous, organic compounds, such as fatty acids, sugars, and other carbohydrates.

**Table 2:** Content of the solutes of effluent harvested from different biodesulfurisation setups

Measurement	PS-1	PS-2	LS	PS-3	FS
pH	9.7	9.7	9.3, 9.4	9.6	9.1
Nitrogen (mgN L^−1^)	<1	3.5	1.5	N/A	6
DNA (mg L^−1^)	N/A	N/A	1–5	1.5	N/A
Organic carbon (mgC L^−1^)	57	24	22	N/A	55

#### 3.4.1 Thiol/disulfides are probable electron shuttling redox couples

There are two ways by which organics could remove sulfide, either by binding sulfide itself^29^ or by acting as an electron sink for the oxidation of sulfide.^30^ Gram-negative bacteria, which are the most abundant bacteria in the biodesulfurisation process,^31^ contain intracellular redox mediators such as glutathione, cysteine, and coenzyme A^32–34^ that might facilitate anaerobic sulfide oxidation. These low weight thiols form a redox couple with their disulfide form, taking up electrons released by sulfide oxidation (eqn (5)) upon breaking the disulfide bond (eqn (6)).

5 H_2_S → S + 2*e*^−^ + 2H^+^

6 2RSH → RSSR + 2*e*^−^ + 2H^+^

In the biodesulfurisation process sulfur is available in overabundance (gr l^−1^), and plenty available for assimilation into thiols. To get an insight in the involvement of thiol/disulfide redox couples as electron sink for sulfide removal we measured the thiol content before and after incubating biomass (∼30 mgN L^−1^) resuspended in solutes in 2.0 mM sulfide for half an hour. During incubation 0.5 mM sulfide was removed. The detection of CoA was hindered due to interference in the CoA peak. We did not observe any glutathione peak in our samples, but we measured an increase in cysteine concentration from 0.06 ± 0.08 to 1.55 ± 0.01 mM during sulfide removal. One mole of cystine can take up two electrons, forming two moles of cysteine. As oxidation of sulfide to sulfur releases two electrons, formation of 1.5 mM cysteine could account for 0.75 mM, enough to explain the observed removal of sulfide.

**Fig. 9:**
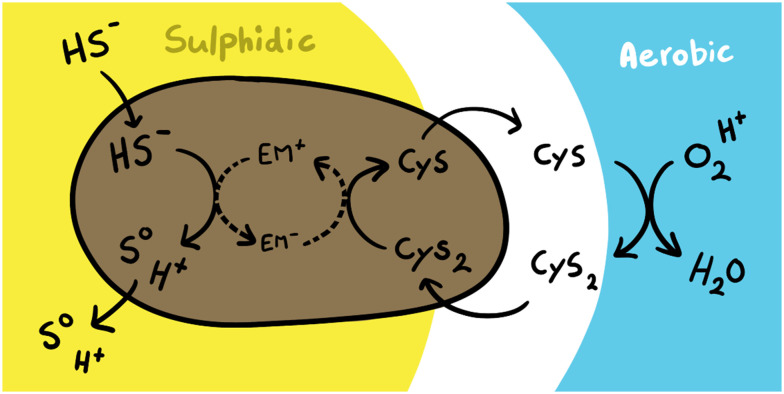
Hypothesised mechanism of electron shuttling by SOB using cystine/cysteine as redox mediator. In sulfidic conditions sulfide is oxidised to sulfur. The electrons go through the cell metabolism, transferring between several electron mediators (EM). Finally, cystine (Cys_2_) is transported into the cell and reduced to cysteine (Cys), which is again excreted. In aerobic conditions the dissolved cysteines are oxidised to cystine.

Cysteine is an amino acid and an easier sulfur source to assimilate into biomass than sulfate, as it needs less reducing power to convert to a suitable redox state. Therefore, bacteria prefer to take up cysteine above sulfate. As in aerobic environments cysteine is quickly oxidised to cystine, bacteria have developed different pathways to transport cystine into the cell.^35^ In *Escherichia coli*, where the intracellular concentration of free cysteine lies around 0.1–0.2 mM,^36^ the uptake of cystine is slightly convoluted. Inside the cell cystine is reduced to cysteine so fast that the intracellular concentration of cystine does not increase and the cystine importer is not inhibited. This results in an import of up to 50 times more sulfur than is required.^37^ Seeing as cysteine is highly reactive and therefore toxic at high concentrations, bacteria have developed several systems for excreting the overload of cysteine,^38^ where it oxidises again when in contact with oxygen outside the cell. The genes for two of the cystine importers employed by *E. coli* are shared among many microbial species,^37^ thus it is possible that this system is also employed by SOB and that SOB excrete high amounts of cysteine. How cystine/cysteine could act as electron shuttlers is visualised in Fig. 9. The electrons released during anaerobic sulfide oxidation are then used to convert cystine to cysteine which is transported out of the cell. This would then explain why the concentration of cysteine in the solutes increased during electron shuttling.

This then also implies that the reactor effluent contains several millimolar of cystine. On a first glance, the shortage of nitrogen would steer away from cystine/cysteine as electron shuttlers. However, the total N measurements were taken after thoroughly aerating the reactor effluent and after all solids were removed from the solution. As during aeration all cysteine would have been converted to cystine, which is poorly soluble in water,^39^ cystine could have partially crystallised and have been removed from the effluent together with all the other solids, resulting in the low dissolved nitrogen content observed. This could explain why adding biosulfur to the solutes of PS-2 induced sulfide removal: the biosulfur pellet would then also have contained cystine crystals.

While we found hints that the cystine/cysteine redox couple is involved, more systematic investigation is needed to confirm its involvement. To confirm the involvement of cystine/cysteine or other disulfide/thiol couples it would be interesting to measure the thiol content of the biomass, biosulfur pellet, and of the solutes before and after incubation in sulfide. Treating samples with TCEP (tris(2-carboxyethyl)phosphine), a strong reducing agent that cleaves disulfide bonds, could give an indication on the degree of reduction of the disulfide/thiol couple.

So far, most investigation on electron shuttling has been performed on mixed SOB cultures. It would be interesting to investigate pure cultures of *Alkalilimnicola ehrlichii* and *Thioalkalivibrio sulfidiphilus*, two species which are thought to be involved in electron shuttling. Transcriptomics could give a glimpse into the metabolism involved in electron shuttling to study if any of the hypothesised routes is present and active.

## 4 Conclusion

We compared the sulfide removal behaviour of five different biodesulfurisation effluents with various sulfide removal capacities. In all effluents biomass was involved in sulfide removal. Sulfide removal in effluent with low sulfide removal capacity could be fully attributed to activity of biomass. But, in effluent that showed medium to high sulfide removal capacities also biosulfur and certain solutes were involved in anaerobic sulfide removal. For reaching the maximal sulfide removal capacity the presence of all three fractions, so biomass, biosulfur, and solutes, were required.

**Fig. 10:**
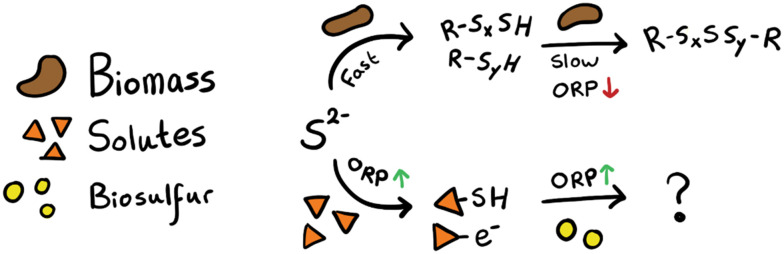
Hypothesised anaerobic sulfide removal mechanisms.

We were able to identify four sulfide removal steps. When we put these patterns in a hypothesised sulfide removal scheme we get Fig. 10. Here, biomass removes sulfide in two steps, a fast chemical equilibrium (as discussed in section 3.2.1) followed by a slow enzymatic conversion (section 3.2.2) where polysulfides are formed and possibly recombined. Parallel to this, sulfide is gradually removed by interactions with certain compounds dissolved in the process solution (section 3.3.1), after which the intermediates are further converted in the presence of biosulfur (section 3.3.2). We hypothesise that dissolved thiol/disulfide redox couples, such as cysteine/cystine, are involved.

By comparing the trends in sulfide removal and ORP of the different effluents and their fractions we discovered that interactions between sulfide and solutes are responsible for an increase in ORP during electron shuttling, contrary to previous believes that this was only caused by the SOB biomass. Electron shuttling biomass was even found to decrease the ORP, underlining that the ORP is a complex term and that the ORP in electron shuttling biodesulfurisation processes need to interpret with care.

## Author contributions

Rikke Linssen: methodology, investigation, formal analysis, writing – original draft, visualisation. Sanne de Smit: supervision, conceptualisation, writing – review & editing. Annemiek ter Heijne: supervision, funding acquisition, writing – review & editing.

## Conflicts of interest

The authors declare that they have no known competing financial interests or personal relationships that could have appeared to influence the work reported in this paper.

## Supplementary Material

EW-012-D5EW00927H-s001

## Data Availability

The data accompanying this work can be found at the 4TU Research Data depository. DOI: https://doi.org/10.4121/b8f6c0bf-8a0d-4392-9081-8af2a18312f2. Supplementary information (SI) is available. See DOI: https://doi.org/10.1039/d5ew00927h.
