# Supplementary material for: Anaerobic sulfide removal involves an intricate interplay between biomass, biosulfur, and solutes
Source: Environ Sci (Camb). 2026 Jan 27;12(3):958–69. doi: 10.1039/d5ew00927h (PMC12839829; doi:10.1039/d5ew00927h)
Supplement: EW-012-D5EW00927H-s001 [file EW-012-D5EW00927H-s001.pdf]

Supplementary Information (SI) for Environmental Science: Water Research & Technology.
This journal is © The Royal Society of Chemistry 2026

SUPPLEMENTARY INFORMATION

Anaerobic sulfide removal involves an intricate interplay between biomass, biosulfur, and solutes

Rikke Linssen [a], Sanne de Smit [a], Annemiek ter Heijne [a*]

[a] Environmental Technology, Wageningen University, P.O. Box 17, Bornse Weilanden 9, 6708 WG, Building Axis z, building nr. 118, 6700 AA Wageningen, the Netherlands. Email address: Annemiek.terHeijne@wur.nl

[*] Corresponding author

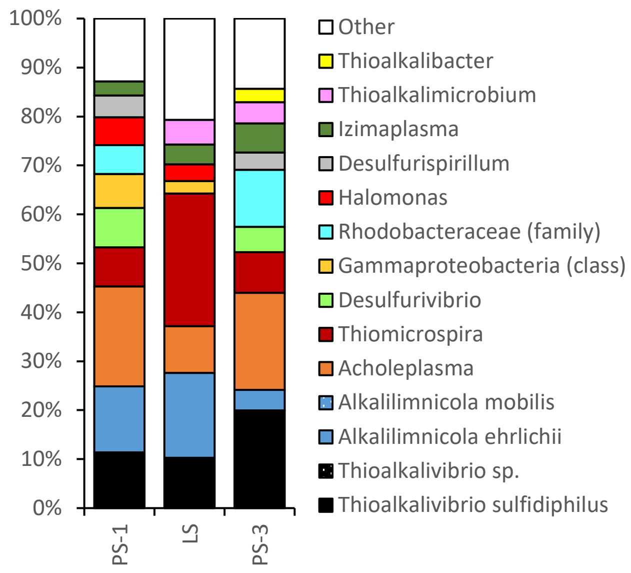

Figure S1 Relative abundance of the SOB communities used in this work. Only relative abundances above 2.5% are shown.

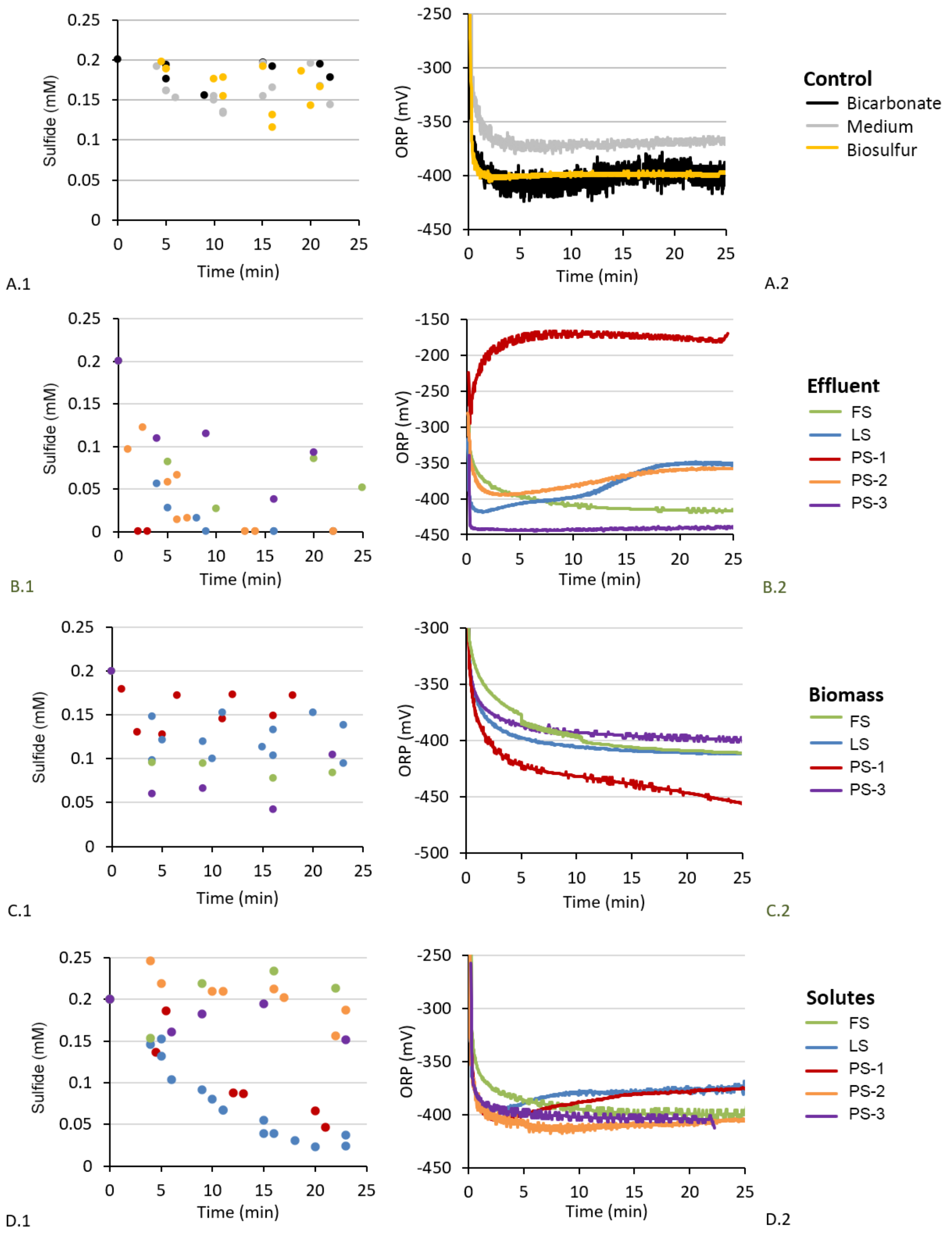

Figure S2 Sulfide concentration (1) and corresponding ORP (2) during anaerobic sulfide removal of different effluent fractions at a sulfide load of 0.2 mM. A) control: bicarbonate buffer (black), reactor medium (grey) and biosulfur in bicarbonate buffer (yellow). B) reactor effluent, C) isolated biomass D) solutes. Different sample sources were used: FS (green), PS-2 (blue), PS-1 (red), PS-2 (orange), and PS-3 (purple).

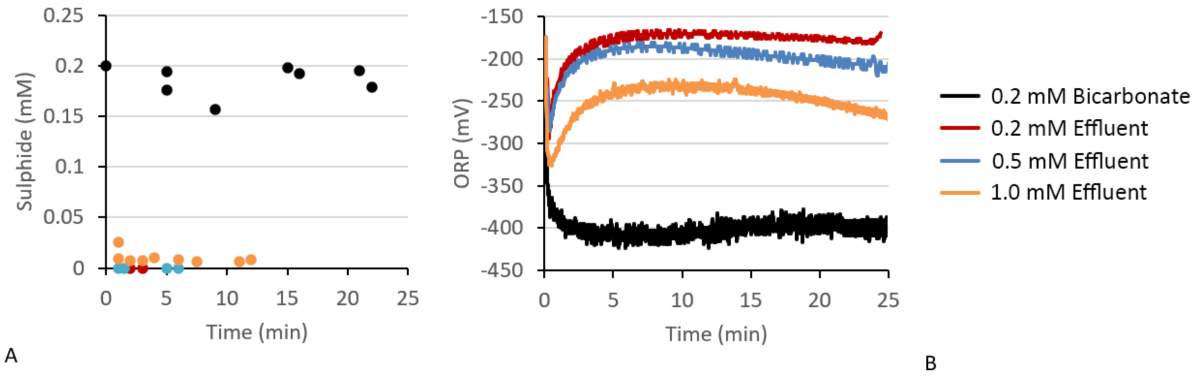

Figure S3 Sulfide concentration (A) and ORP (B) during the sulfide removal experiment with PS-1 effluent and an anaerobic control (black). Sulfide dosing was 0.2 mM (red), 0.5 (blue), and 1.0 mM (orange).

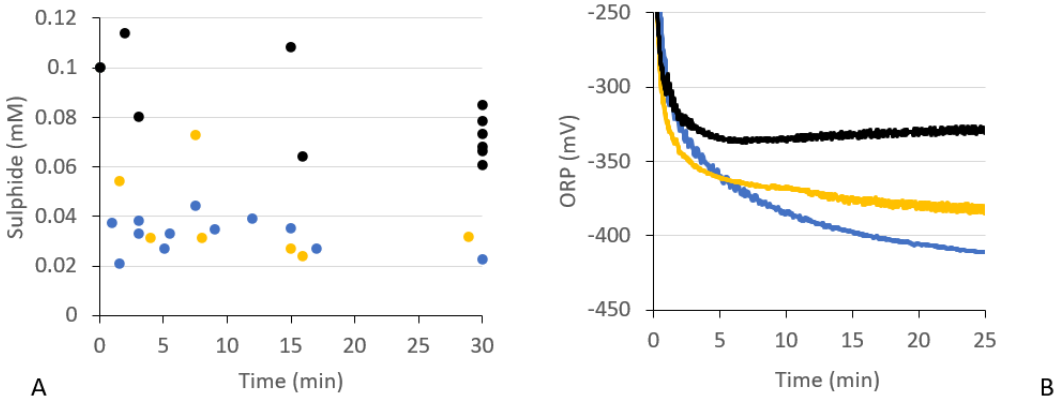

Figure S4 Sulfide concentration (A) and ORP (B) of LS (yellow) and PS-3 (blue) biomass or abiotic control (black) dosed with 0.1 mM sulfide.

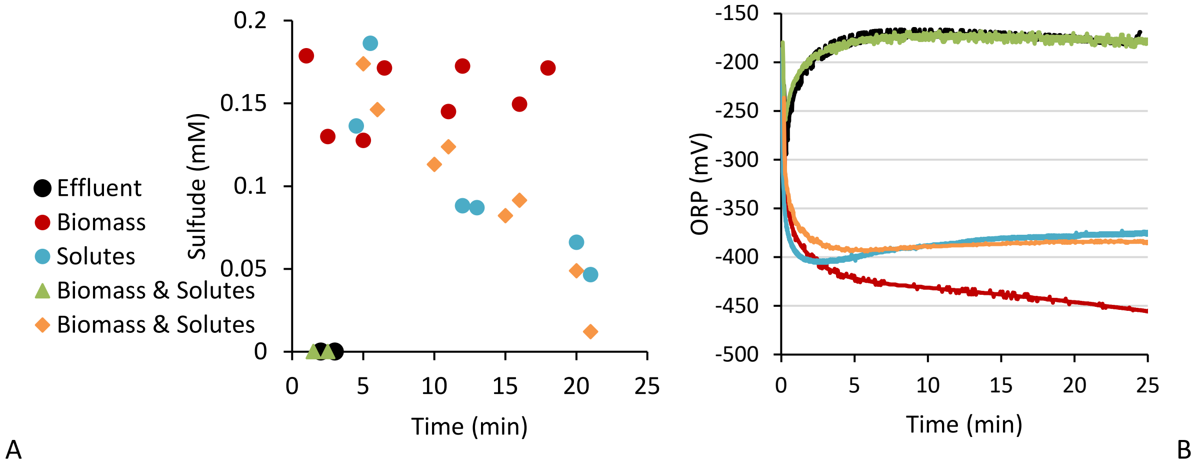

Figure S5 Sulfide concentration (A) and ORP (B) during anaerobic sulfide removal of (combined) fractions of PS-1 effluent. The sulfide load was 0.2 mM. Biomass was recombined with solutes either before (green, triangles) or after (orange, diamonds) overnight aeration.

Table S1 The ORP and sulfide removal patterns observed during different anaerobic sulfide removal processes in effluent of several biodesufurisation setups. Different fractions (full effluent, biomass, biosulfur, and solutes) or combinations were investigated. The timeframes during which the processes occurred are listed per effluent source and fraction. If a timeframe is not mentioned, said fraction or combination was not tested.

**Pattern**	**Effect on ORP**<br>***Sulfide removal***	**Fraction or combination**	**PS-1**	**PS-2**	**LS**	**PS-3**	**FS**
1	No ORP effect<br>*Partial sulfide removal*	Biomass<br>Biomass + S^0	<5	<5<br><5	<5	<5	<5
2	Gradual (rapid) increase in ORP<br>*Constant sulfide removal*	Effluent<br>Solutes<br>S^0 + solutes	N.d.<br>2-15	2-7<br>N.d.<br>2<	2-10<br>2-10	N.d.<br>N.d.	N.d.<br>N.d.
3	Gradual increase of ORP<br>*Sulfide depleted*	Effluent<br>Biomass + solutes	1-5<br>1-5	2-20<	10-20	N.d.	N.d.
4	Constant decrease in ORP<br>*No sulfide removal / sulfide depleted*	Effluent<br>Biomass	15<<br>1<	N.d.<br>1<	 1<	N.d.<br>1<	N.d.<br>N.d.

N.d. this process was not detected

SI A. Other (preliminary) studies performed on identifying solutes

Aside from the data shown in Table 2 in the main text, additional experiments were performed with the goal of identifying or characterising the solutes present in the effluent. To do this, COD was measured, ICP was performed, and NMR was tried.

COD

The chemical oxygen demand (COD) of the solutes was measured (LCK114, Hach, US) and were the following (in mgO_2/L): PS-1 310, PS-2 360, PS-3 170, LS 1190, FS 200). The COD does not seem to correlate with capacity for sulfide removal of the solutes. COD is influenced by all compounds that can be oxidised, including organic carbons and thiosulfate. As biodesulfurisation effluent often contains thiosulfate (De Rink et al., 2021) but the thiosulfate content was not measured, the COD had low experimental value.

ICP

ICP was performed on PS-1 solutes with and without the addition of 4 mM sulfide. After an hour incubation in anaerobic conditions, the sample was measured with and without acidic digestion pretreatment. Digestion was performed using acidification and heating. 0.5 gram of sample was mixed with 7.5 ml hydrochloric acid and 2.5 ml nitric acid (Aqua Regia) into 100 ml closed Teflon vessel. The samples were heated using microwave (Milestone Ethos 1 rotor HPR 1000/10) using the following program: In 5 min ramp to 100 C; from 5 to 10 min ramp to 130 C; from 10 to 15 min ramp to 180 C; remain 180 c until 30 min; 10 min cooling down. The total heading time was 40 minutes. At maximum 1400 Watt is used. Afterwards vessels were cooled down for another hour in the fume hood before opening them. Vessel content was transferred into 100 ml milli-Q water. Then 10 ml of digested and undigested samples and standards were acidified with 1% HNO3 (v/v) and measured by ICP (Avio 500 ICP Optical Emission Spectrometer, PerkinElmer). The following parameters were used: A nebuliser from Meinhard type K with a gas flow of 0.7 L/min, an auxiliary gas flow of 0.2 L/min, a baffled glass cyclonic spray chamber, a sample uptake rate of 1.0 mL/min, a RF power of 1500 W, a 2.0 mm id alumina injector. Calibration lines ranged from 20 u/L-100 ug/L, 200-1000 ug/L and 2000-10,000 ug/L depending on the measured elements.

The idea was that sulfide would escape the liquid during digestion, and that bound or oxidised sulfide would be measured with ICP. This would then show if sulfide was converted to elemental sulfur or to organosulfurous compounds. However, the solutes itself already contained too much S (130 mM), likely in the form of sulfate and thiosulfate, to see a significant change in S after sulfide addition.

NMR

NMR can be used to identify or characterise various compounds. Therefore, the solutes were scanned using 1H-NMR (Spinsolve 80 Ultra, Magritek, Germany). Between 32 and 512 scans were performed per solutes sample at an acquisition time between 3.2 and 6.4 seconds and a repetition time of 15 seconds. The signal of the aqueous solution (the water peak around 4.8 ppm) was supressed so peaks close to the water peak were more visible. Nevertheless, no spectra were obtained.
